# *Candida* spp. in Human Intestinal Health and Disease: More than a Gut Feeling

**DOI:** 10.1007/s11046-023-00743-z

**Published:** 2023-06-09

**Authors:** Irini A. M. Kreulen, Wouter J. de Jonge, René M. van den Wijngaard, Isabelle A. M. van Thiel

**Affiliations:** 1 Tytgat Institute for Liver and Intestinal Research, Amsterdam Gastroenterology, Endocrinology and Metabolism, Amsterdam UMC, Location Academic Medical Center, Meibergdreef 69-71, 1105 BK Amsterdam, the Netherlands; 2 Department of Gastroenterology and Hepatology, Amsterdam UMC, Location Academic Medical Center, Meibergdreef 9, 1105 AZ Amsterdam, the Netherlands; 3 Department of General, Visceral, Thoracic and Vascular Surgery, University Hospital Bonn, 53127 Bonn, Germany; 4 Royal Netherlands Academy of Arts and Sciences, Westerdijk Fungal Biodiversity Institute, Uppsalalaan 8, 3584 CT Utrecht, the Netherlands

**Keywords:** Inflammation, Mycobiome, Microbiome, Preterm, Colonization, Strain diversity

## Abstract

Fungi are an essential part of the normal collection of intestinal microorganisms, even though their collective abundance comprises only 0.1–1% of all fecal microbes. The composition and role of the fungal population is often studied in relation to early-life microbial colonization and development of the (mucosal) immune system. The genus *Candida* is frequently described as one of the most abundant genera, and altered fungal compositions (including elevated abundance of *Candida* spp.) have been linked with intestinal diseases such as inflammatory bowel disease and irritable bowel syndrome. These studies are performed using both culture-dependent and genomic (metabarcoding) techniques. In this review, we aimed to summarize existing data on intestinal *Candida* spp. colonization in relation to intestinal disease and provide a brief overview of the biological and technical challenges in this field, including the recently described role of sub-species strain variation of intestinal *Candida albicans.* Together, the evidence for a contributing role of *Candida* spp. in pediatric and adult intestinal disease is quickly expanding, even though technical and biological challenges may limit full understanding of host-microbe interactions.

## The Human Gut Mycobiome

The human intestinal tract is home to an abundant collection of microorganisms, estimated at a size of approximately forty trillion cells [1]. While the vast majority of microbes consists of bacteria, the fungal compartment has in recent years also been shown to contribute to health and disease. It is estimated based on genomic techniques that fungi comprise only 0.1% of the total microbial community [2], but due to their larger physical size and metabolic activity it is nevertheless likely that these organisms take part in (patho)physiological processes. For example, intestinal fungi contribute to the development of systemic and mucosal immunity. To illustrate this concept, it was shown that fungi-depleted mice show worsened disease in models of intestinal or airway inflammation, and that the general immunity including IgG antibodies were increased under the influence of gut fungi (colonization) [3, 4], as also reviewed by Gutierrez et al. [5]. Knowledge regarding their role in human intestinal disease is accumulating, but unfortunately, much less is known about the fungal gut composition than the bacterial composition.

**Fig. 1:**
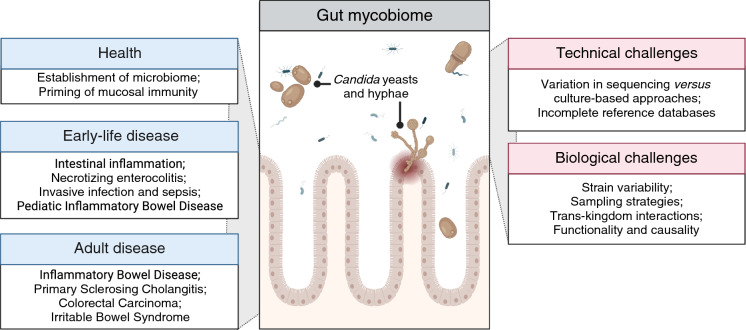
Schematic overview of this review article. Here, the gut mycobiome in relation to health and (pediatric) disease is described, as well as challenged faces in the field of mycobiome research. Illustration created with https://BioRender.com

In humans, the composition of the gut mycobiome changes with age, but in general yeasts are more commonly observed than filamentous fungi at any age in studies regarding the fecal or intestinal mycobiome [6, 7]. Based on culture-dependent and -independent techniques, species within the genera *Candida* and *Saccharomyces* are especially often described in fecal samples. Since *Candida* spp. are classified both as commensal and as opportunistic pathogens, research often involves the role of *Candida* spp. in the intestine. In addition, with more than 150 species [8] and varying levels of relatedness between species [9], the genus is very diverse and thus has varying levels of possible pathogenicity and relevance to (intestinal) disease. In this review, we aim to summarize current knowledge regarding colonization during human lifetime and abundance of the most common *Candida* species in the gastrointestinal tract with a predominant focus on inflammatory and functional intestinal disease (Fig. 1). In addition, we will address challenges in studies focused on the gut mycobiota.

### Early-Life *Candida* spp. Colonization of the Intestine

The important role of the intestinal (bacterial) microbiome for human health and disease has become more clear last decades. Similarly, fungall intestinal colonization already starts during the first days of life until 6 months of age, as fungal signatures have been found in intestinal-derived biological specimens such as feces and anal- and rectal swabs. A general characteristic of the early-life mycobiota appears to be a low fungal richness during the first months of life [10, 11]. Furthermore, Rao et al*.* described inconsistent fungal loads that did not correlate with the age of preterm neonates during the first 6 postnatal weeks, all in contrast to predictable bacterial dynamics over time [12]. Taking these characteristics into account, early-life studies describe *Candida* spp., *Saccharomyces* spp., and *Debaryomyces* spp. as most common genera in intestinal-related samples [10, 11]. Schei et al*.* determined mycobiome profiles of 298 neonate-mother pairs from birth until 2 years of life using 18S and ITS1-based metabarcoding, in which the genus *Debaryomyces* was most often detected during the period of breastfeeding. After weaning, i.e., the phase when solid foods are introduced, *Saccharomyces cerevisiae* abundance stabilized [11]. Notably, and in contrast to other studies, this study showed low abundance of *Candida albicans* in fecal samples of the neonates. The recent study from Ward et al. determined ITS2-based mycobiome profiles of 17 neonates during the first 30 days of life in parallel with anal samples of 16 mothers. The most abundant *Candida* species in the infant anal samples were *Candida parapsilosis, Candida tropicalis,* and *C. albicans* (relative abundance levels of respectively 32.24, 23.8, and 15.1 per cent) [10]. Also Kondori et al. observed presence of *C. albicans* and *C. parapsilosis* in feces and rectal swabs through a culture-based method in 133 Swedish neonates. Besides, several smaller studies that included preterm neonates also describe *Candida* spp. as most prevalent in fecal samples [13–15]. In conclusion, fungal colonization seem to occur already during the first few weeks after birth and is predominantly defined by presence of *Candida* spp.

The majority of the early-life studies are performed on longitudinal samples collected from birth until the first months of life, and focus on colonization and route of birth. Strati et al*.* described the ITS1-based colonization dynamics in feces from 57 separate individuals (divided over early life, childhood, adolescence, and adult (mean 12 ± 9.5 years of age) [16]. Fungi were detected in 80% of the individuals, of which *C. albicans* was most commonly observed, followed by *Rhodotorula mucilaginosa* and *C. parapsilosis*. To gain a more detailed insight in the development of the fungal gut community, there is a clear need for longitudinally collected samples per individual, as well as increased study cohort sizes. This would subsequently allow for understanding of the dynamics and stabilization related by age, and how environmental factors, diet, and medications contribute to this process. Nevertheless, detection of increased abundance of *Candida* spp*.* shows strong evidence of its important contribution to early-life mycobiome colonization.

### The Adult Intestinal Mycobiome is Regularly Dominated by *Candida* spp.

Various studies have described the composition of the adult mycobiota, even though mostly in relation to the diseased individual. Based on both DNA- and culture-based approaches, the five most commonly found species in the adult gut mycobiome are, in decreasing order of relative abundance, *C. albicans*, *S. cerevisiae*, *C. tropicalis*, *C. parapsilosis*, and *Candida glabrata* [6]. Recent studies, albeit with relatively small sample sizes, have since obtained similar results being that *Candida* spp. together with *Saccharomyces* spp. dominates the healthy gut mycobiome [17–25]. In contrast to the smaller studies, two larger cohorts have recently been investigated. One study of 1244 Danish middle-aged and elderly individuals once again confirmed predominant abundance of *Candida* spp. as determined by ITS2-based metabarcoding [25]. However, in a description of the fungal compartment of the Human Microbiome Project (HMP) (*N* = 147), *Candida* spp. ranked only as third most abundant genus after *Saccharomyces* spp. and *Malassezia* spp. [26]. Sequences referring to *C. albicans* were described in 80.8% of all samples. For this cohort, longitudinal samples were available, and *C. albicans* was detected at both time points in 63.6% of the individuals. Thus, while *Candida* spp. was not the most abundant genus in this study, its presence is well established within the human fecal mycobiome.

The vast majority of studies regarding intestine microbes focuses on the fecal profile of bacteria or fungi. However, it is now understood that the luminal (fecal) bacterial community is very dissimilar to the fraction of fungal cells that actually directly associate and interact with the intestinal mucosal tissue. Unfortunately, only a few studies have focused on the (healthy) mucosa-associated fungal counterpart. Recently it was shown that the murine luminal gut mycobiome and mucosa-associated fungal composition are indeed very different [27]. In this murine setting, *Candida* spp. was more abundantly present in the mucosal samples than in stool samples. For human samples, it was shown that these have abundance of *Candida* spp. [27, 28]. Taken together, *Candida* spp. is often the most dominant genus described in intestine-related samples. Unfortunately, the vast majority of metabarcoding studies does not classify the yeast species beyond the genus level, leading to a lack of detail on species abundance classification in the fecal mycobiome. Based on previous (review) articles, and in combination with the known pathogenic potential of several members of the genus *Candida*, we henceforth mainly focus on *C. albicans, C. parapsilosis,* and *C. glabrata* in the intestinal tract.

## The Role of *Candida* spp. in Intestinal Disease

While the gut fungal community contributes to normal development of immune responses, also within the mucosal barriers of the intestine, evidence for a contributing role of the gut mycobiome in gastrointestinal disease is accumulating. In this section, we will review current research regarding *Candida* spp. in relation to prematurity and the associated intestinal inflammatory disease necrotizing enterocolitis (NEC). Moreover, we will briefly touch upon candidiasis before moving focus to (adult) intestinal diseases such as inflammatory bowel disease, colorectal carcinoma, and irritable bowel syndrome.

### *Candida* spp. in Pre-term Infants

As described before, *Candida* spp. are important players in mycobiome colonization. However, in complex early-life situations such as preterm birth (< 37 weeks of gestation) the intestine and immune system are still immature. In these instances, altered microbial profiles can contribute to acute life-threatening complications that even have long lasting impact later in life, such as atopic, allergic, and metabolic diseases [15, 29, 30]. Factors that influence the microbiome composition in early life are gestational age, hospital-associated microbiome, feeding regimes and clinical practice, such as antibiotic treatments [30, 31]. Broad-spectrum antibiotics are used frequently in preterm neonates to prevent disease by pathogenic bacteria. However, this use is described to increase susceptibility of preterm neonates for fungal overgrowth and an increased risk of horizontal transmission of hospital-associated fungi [30, 32]. Especially extreme low-birthweight neonates (< 1000 g) were at risk for development of candidiasis when treated with certain broad spectrum antibiotics, as reviewed by Kilpatrick et al. [33].

Multiple studies aimed to characterize the intestinal microbial composition of preterm neonates, in which *Candida* spp. abundance was often described. Henderickx et al*.* recently determined the fecal mycobiome profiles of 50 preterm neonates (24–36 weeks of gestation) and in 6 term neonates (37–40 weeks) during the first 6 weeks of life [13]. ITS2-based metabarcoding analysis showed predominant abundance of *Candida* spp. species during these first 6 weeks and the abundance increased with gestational and post-natal age [13], in line with earlier research by Rao et al. [12]. The enrichment of *Candida* spp. was mostly seen in vaginally born preterm neonates, while C-section delivery showed more enrichment for the skin related fungal genus *Malassezia* [13]*.* To accelerate the microbiome maturation in these infants, Samara et al. assessed the effects of multi-strain (bacterial) probiotics in 57 preterm neonates (i.e., < 1000 g birthweight and < 29 weeks of gestational age) in a randomized control trial. Both bacterial and fungal fecal communities were analyzed before, during, and 6 months after probiotic treatment using 16S and ITS2-based metabarcoding methods respectively [31]. Probiotic treatment induced a significant reduction of the relative abundance of *Candida* spp. compared to neonates that did not receive probiotics [31]. A smaller study by James et al. described the mycobiome of 11 preterm neonates (25–36 weeks of gestation) over a period of 6, 12, and 18 months after birth, using ITS1-based metabarcoding [15]. Although a low fungal diversity was described until the first 6 months, multiple genera were detected. *Candida* spp., *C. albicans, C. metapsilosis, C. parapsilosis,* and *C. tropicalis* were detected in all samples with a large range of abundance levels, varying from 0.02–97.1% over the first 12 months of life. Gestational age was predominantly driving the distribution of *Candida* spp. abundance. The prevalence and abundance of *C. parapsilosis* specifically was higher in the six youngest preterm neonates (< 31 weeks of gestation) compared to the older 5 preterm neonates (> 31 weeks of gestation). Abundance of *C. albicans* was much higher in the five oldest neonates compared to the youngest group. Because the data of *Candida* spp. abundance in (preterm) neonatal fecal mycobiome is highly variable, it is important to take the small sample numbers into account. Further research should focus on larger cohorts and longitudinally collected samples to determine less variable mycobiota profiles during (pre-term) early life.

#### Intestinal Inflammation and Enterocolitis

Preterm neonates are at risk for development of systemic infections and intestinal inflammatory diseases. Necrotizing enterocolitis (NEC) is the most common acute neonatal intestinal disease which occurs in 5–10% of preterm neonates and hereby is the leading cause in morbidity and mortality in the neonatal intensive care units (NICUs). Although a clear disease definition remains to be established, important features are the presence of gas within the intestinal wall (pneumatosis intestinalis), inflammation, and an altered microbiome composition [34, 35]. In addition, systemic infections such as sepsis are also occurring frequently in preterm neonates, even in combination with NEC. Sepsis is defined as a life-threatening organ dysfunction caused by a dysregulated host response to infection [36]. Although NEC is often associated with bacterial pathogens, Stewart et al. studied the fecal mycobiome colonization in preterm neonates (< 32 weeks; *N* = 32) by using 28S rRNA PCR followed by denaturing gradient gel electrophoresis (DGGE) to identify individual species. In this study, seven preterm neonates developed NEC and thirteen individuals developed sepsis as diagnosed based on positive blood cultures [37]. Non-viable fungi were detected in stool of 3/8 sepsis patients, from which *C. albicans* and *C. glabrata* were most abundant (respectively 30% and 29%). However, no fungi were detected in stool of NEC patients, which is probably due to administration of the antifungal fluconazole [37]. A recent retrospective study of Garg et al. described the occurrence of sepsis in neonates with NEC (*N* = 209 neonates) determined by positive blood cultures, and showed that 23.9% eventually developed sepsis [38]. Of these cases, 6.7% were diagnosed with *Candida*-mediated sepsis. Further assessment indicated that both *C. albicans* and *C. parapsilosis* was found in blood cultures of these patients. Another severe complication of NEC is intestinal perforation, although the latter may also occur spontaneously (i.e., NEC-independent) as is the case for focal intestinal perforation (FIP). Coates et al. compared 80 NEC patients with 36 FIP neonates in a retrospective cross-sectional study [39]. Of the FIP patients, 44% had positive *Candida* blood cultures compared to 15% of the NEC patients.

Related to NEC is another form enterocolitis, namely that associated with Hirschsprung’s disease. In this congenital disease that affects 1:5000 newborns, the distal colon lacks ganglion cells and children therefor cannot expel fecal matter. The resulting bowel obstructions increases the risk for enterocolitis development. Frykman et al*.* determined the mycobiome profile of Hirschsprung patients with (*n* = 9) and without (*n* = 9) enterocolitis development with ITS1-based metabarcoding analysis [40]. The mycobiome of infants with Hirschsprung-associated enterocolitis (HAEC) was lower in diversity compared to that of Hirschsprung without enterocolitis. The authors observed two different HAEC gut mycobiota profiles based on abundance of *C. albicans*. The ‘high-burden’ group had high abundance of *C. albicans* (97.8%) and low *C. tropicalis* abundance (2.2%), while the ‘low-burden’ group had *Candida* spp. levels comparable to non-HAEC patients. Whether this is directly related to development of enterocolitis cannot be determined from this study.

#### Invasive *Candida* Infection

Invasive candidiasis is one of the other common causes of mortality in extremely preterm neonates, in which a positive *Candida* culture is determined in sterile body fluids such as blood, cerebrospinal fluid and urine [41]. A retrospective multicenter study in the US, performed in 2004–2007, described a 9% rate of candidiasis among this population. A more recent retrospective study of Warris et al*.* studied pediatric candidiasis in a multicenter European project between 2005 and 2015 [42]. 36.4% of the sepsis cases occurred in neonates and were related to higher mortality rates at the NICUs. *C. albicans* caused 60.2% of the candidiasis cases in the neonatal patients. Another important observation was the significant difference in *C. albicans* related candidiasis in Northern European centers versus higher *C. parapsilosis* caused infections in Southern centers.

Despite the limited number of studies available, *Candida* spp. are important commensals in the neonatal intestine, but in immunocompromised individuals, as is the case for premature neonates, *Candida* spp. may cause and contribute to life-threatening intestinal and inflammatory diseases in early life. Although risk factors such as low birth weight, prolonged hospitalization, use of catheters, and skin carriage of microbes are becoming more clear, there is need to improve understanding how the *Candida* spp. interact with the surrounding microbiome and neonatal host. Unfortunately, research regarding preterm and neonatal conditions are only sparsely performed due to the complicated in vivo and in vitro models. Evolvement of models would provide opportunities to interfere and to prevent and treat vulnerable neonates from *Candida* related disease.

### Inflammatory Bowel Disease

Inflammatory bowel disease (IBD) is characterized by chronic inflammation of the gastrointestinal tract, and Crohn’s diseases (CD) and ulcerative colitis (UC) are the most common subtypes of IBD [43, 44]. Symptoms generally include abdominal pain, diarrhea, weight loss, and presence of blood in stool. In the research field of gastroenterology, this disease is commonly divided in pediatric and adult disease, and we will here maintain this separation. It is thought that the interplay of genetics, environmental stressors, and microbial interactions all contribute to dysregulated immune responses [45]. The important role of intestinal fungi in (pediatric) IBD is currently being uncovered.

#### Pediatric IBD

Early onset of IBD occurs already before the age of 10 years [46] and occurs in approximately 9–10 individuals per 100.000 in Europe [47]. A recent meta-analysis showed an alarming sevenfold increase for pediatric CD over the past 50 years. The incidence of UC increased as well, but slightly less with a factor of 3.5-fold [47]. Alterations of the bacterial component in the gut of pediatric IBD (pIBD) patients have been described in several studies, though a recent systematic review by Zhuang et al. on microbiota profiles in pediatric IBD did not identify a consistent specific gut microbiota for pediatric IBD [48]. The lack of consensus was presumably due to heterogeneous methodologies and small patient cohorts. Investigations regarding the fungal mycobiome in relation to pIBD are very scarce, and not much is known yet about their role in pediatric disease.

It is hypothesized that intestinal fungi play an important role in the inflammation status of immunocompromised patients. A few pIBD studies have been performed to characterize the mycobiota related to the this disease, in which low diversity and altered fungal composition are described compared to non-IBD control individuals. Chehoud et al. compared ITS1-based metabarcoding analysis of fecal mycobiota profiles using 32 pediatric patients *versus* 90 non-IBD (pediatric and adult) individuals [49]. A significantly lower Shannon index was determined in the pIBD samples and these samples also clustered separately from the control group. Elevated abundance of the *Saccharomycetales* taxa was observed, mainly attributed to increases in *Candida utilis* (presently named *Cyberlindnera jadinii*) and *C. parapsilosis* abundances [49]. Notably, sequences belonging to the latter species were almost exclusively detected in pIBD patients. Abundance of *C. parapsilosis* also correlated positively with *Bacteroides fragilis* [49], an enterotoxic bacterium that has been linked to active CD [50]. This finding may contribute to understanding of inter-kingdom interactions and the immunogenic capacity of the microbial community. A second study focused on the microbial composition in pIBD and described elevated abundance of *C. albicans* [51]*.* However, only five fungal taxa were described in this DNA-based analysis, and it is thus likely that a re-analysis of these samples through newer protocols would indicate more (differentially abundant) taxa [51]. Another a study was performed on newly diagnosed, treatment naive Saudi Arabian patients with CD (*n* = 15) and on non-IBD control infants (*n* = 20) [52]. ITS metabarcoding was performed on both mucosal tissue samples and fecal samples. After *Ophiosphaerella agrostis* and *Hanseniaspora uvarum,* the abundance of *C. parapsilosis* was detected as third most differentially abundant fungal taxon in inflamed *versus* non inflamed mucosal tissue, albeit no significant difference was found. The fungal mycobiome detected in the fecal samples did show increased significant abundance of non-*Candida* spp. taxa. Also Mukhopadhya et al. investigated the fungal mycobiome of 25 pIBD patients compared to 12 non-IBD control individuals [53]. Fungal loads and 18S sequencing profiles were determined on biopsies taken from the distal colon. Basidiomycota were more dominant in pIBD samples compared to non-IBD controls. Since 18S sequences have a limited capacity of discrimination of lower taxonomic levels, we suggest that other techniques should be used to confirm presence of fungal genera and species such as *Candida* spp. in these precious pIBD samples [54]. Most recently, Ventin-Holmberg et al. assessed fecal samples of 30 pediatric IBD patients to determine ITS-based mycobiota profiles before and during anti-tumor necrosis factor alpha (TNFα) treatment, a key biological treatment in IBD [55]. Half of the patients responded to the infliximab treatment, and non-responders showed increased *Candida* spp. abundance and specifically an 38% increase of *C. albicans*. In contrast, responders to infliximab showed increased abundance of *Saccharomyces* spp. This might indicate an important role for *C. albicans* in relation to pIBD development and persistence [55].

In conclusion, a clear mycobiome composition for pediatric IBD has not been characterized yet, but reduced diversity and altered mycobiota profiles with a role for *Candida* spp. seem to be common denominators. These findings provide important insights in the role of the mycobiome in pediatric IBD. However, functional effects of these signatures on pediatric IBD development remains to be explored.

#### Adult IBD

Inflammation of the intestines has a largely similar symptomatic presentation for both children and adults, although the specific disease locations can vary [56]. The incidence of adult IBD is 0.3% worldwide, but these diseases are more often diagnosed in Western countries [57]. As indicated before, the role for the gut bacterial community is quite extensively described in IBD, and the notion that luminal microbes are involved in intestinal inflammation is now widely accepted. For the involvement of the fungal component specifically, we kindly refer to the recent review by Underhill and Braun [58].

In patients with IBD, the diversity of the fungal community is, in general, lower than that of healthy volunteers. Furthermore, the abundance of *Candida* spp. or *C. albicans* is oftentimes found to be elevated [18, 20, 59]. Only a few studies have touched upon the mucosal community of fungi, but these results are partially contrasting. In a study by Liguori et al., *C. glabrata* was more abundant in mucosal samples of CD patients, but the genus *Candida* was not differentially abundant [60]. According to Limon et al., *Candida* spp. was the most abundantly present genus, both in healthy volunteers and IBD patient mucosal samples, but again the abundance of *Candida* spp. was not significantly different between the two populations [59]. Last, it was recently described that *Candida* spp. was also more abundantly present in mucosal lavage samples of IBD patients [28]. Taken together, the existing data on the fecal and mucosal communities generally indicates that a higher abundance of *Candida* spp. is observed, and that this is associated with inflammation of the intestine.

Multiple lines of evidence support the importance of gut fungi in IBD, including the signaling pathways responsible for fungal recognition and control in case of apparent infection. Early genome-wide association studies (GWAS) in IBD described single nucleotide polymorphisms in various elements of the most important fungal-related signaling pathway. In brief, fungal cell wall antigens can be recognized by the membrane receptors Dectin-1, Dectin-2, and Mincle. The activation signals are carried on downstream through spleen tyrosine kinase (SYK) and further downstream through CARD9. Especially the latter is associated with intestinal inflammation, as one of the polymorphisms (CARD9^S12N^) is associated with an increased risk for both CD and UC [61]. The aforementioned review extensively described additional genetic variants associated with IBD in the antifungal recognition pathways [58].

Signaling processes underlying the involvement of fungi in IBD are often evaluated in murine models of intestinal inflammation. A commonly used model to investigate the involvement of the intestinal myeloid compartment is in the dextran sodium sulfate (DSS) model. Mice receive oral DSS which chemically disrupts the intestinal epithelial lining and subsequently allows for exposure to luminal microbes and their metabolites. Iliev et al. have used this model in combination with defective anti-fungal immunity by means of *Dectin-1* knockout mice [62]. Knockout mice receiving DSS had far worse disease, and fecal *C. tropicalis* abundance expanded in *Dectin*-*1*^−/−^ mice upon DSS exposure. In multiple follow-up experiments using DSS-colitis, the authors show that proper antifungal recognition is necessary to limit the exacerbating effect of fungi in the murine intestine [62]. In addition, colitogenic mice also showed signs of humoral immune responses, as the levels of anti-*S. cerevisiae* antibodies (ASCAs) in serum of these mice was elevated. These antibodies recognize mannans, and studies indicated that cell wall structures of *C. albicans* are a likely epitope of these antibodies as well [63, 64]. Interestingly, these antifungal antibodies are often detected in patients with CD, but less for UC [65]. Recent work by Doron et al. has shown that the gut mycobiota are indeed responsible for induction of a multitude of antibodies. These antibodies were especially directed against the hyphal form of *C. albicans*, leading to the insight that more virulent yeasts would more potently induce antibody responses. The authors suggest that this process is dysregulated in the context of CD [66]. These recent discoveries highlight the complexity and heterogeneity of not only intestinal diseases, but also the challenges in host-mycobiota interactions. In conclusion, the gut mycobiome of IBD patients is altered, but the exact manner through which gut fungi influence (severity of) the diseases remains to be further investigated.

With multiple aspects of fungal involvement in IBD being partially elucidated, modulation of the gut mycobiome is occasionally investigated as remedy for intestinal disease. Oftentimes, these therapies aim to restore altered ‘disease-associated’ microbial communities to resemble that of healthy individuals, and a thorough way to do so is through fecal microbiota transplantation (FMT). In such experimental treatment settings, feces of a healthy individual is processed and administered to the diseased individual through nasogastric tube, endoscopy, or a combination hereof. Unfortunately, the success rates of this type of treatment is relatively low. However, Leonardi et al. investigated IBD fecal samples of recipients of FMT, and observed that high abundance of *Candida* spp. pre-FMT correlated with a response to this treatment [20]. This observation was later confirmed in another cohort [23], indicating that a certain fungal (and total microbial) composition could indeed possess pro-inflammatory capacities. Furthermore, this notion opens the possibility to screen patients for FMT for certain microbial compositions that associate with response, or allow for specific anti-*Candida* treatment in IBD.

### Colorectal Carcinoma

Patients with IBD have a higher chance of developing colorectal carcinoma (CRC) due to prolonged inflammatory processes and disturbances. In the general population, the incidence of colorectal cancers is approximately 4% and thereby among the most prevalent cancers, but the risk for IBD patients to develop CRC is approximately 2-to-threefold higher [67]. A plausible role for the luminal gut fungi composition was mainly described in two sets of back-to-back publications. The first publications indicated that the IBD-susceptibility gene CARD9 was also associated with cancer development as shown in a murine model of CRC [68, 69]. Both studies showed that Card9-deficient mice were not only more susceptible to chemically induced colitis as described above, but also developed a higher tumor burden upon challenge with the carcinogenic agent axozymethane (AOM) followed by DSS (AOM–DSS). In addition, Wang et al. observed elevated abundance of *C. tropicalis* in *Card9*-deficient mice, and feeding mice with this yeast increase the tumor burden in the AOM–DSS model, likely through the induction of myeloid-derived suppression cells that exert immunosuppressive properties in the tumor environment [69]. In relation to this finding, the authors describe that patients with CRC have an elevated abundance of *C. tropicalis* and a decreased abundance of *C. albicans.* In addition, the fungal load of the investigated tumors correlates with the number of myeloid-derived suppressor cells (MDSCs) in blood and colon of patients with CRC. These MDSCs actively dampen the local immune environment and hence allow for less restricted tumor growth. Together, these two papers indicate that proper antifungal signaling is protective against tumor development in the AOM–DSS mouse model of CRC, and that *C. tropicalis* may be related to cancer progression.

Two recent studies assessed previously generated metagenomic sequencing of The Cancer Genome Atlas (TCGA) data to determine tumor-associated mycobiota [70, 71]. Although these studies were originally aimed at description of the bacterial composition, the authors were able to extract fungal data, and both articles describe the fungal communities associated with multiple tumor types. In both studies, presence of *C. albicans* with particularly high abundance in the colon. Dohlman et al. additionally describe abundance of *C. tropicalis* [71], and Narunsky-Haziza et al. mention presence of *C. parapsilosis* [70]. In the prior study, transcriptionally active *Candida* was determined to be predictive of worse survival for gastrointestinal cancers. The authors suggest that *C. albicans*-mediated inflammation dysregulates epithelial homeostasis and hence could stimulate formation of metastasis [72]. In contrast to these two publications, a similar study focused on CRC alone does not describe (elevated) abundance of *Candida* spp. [72]. Taken together, further investigations should point out whether intestinal fungi, and specifically *Candida* spp. have a protecting effect against tumors, or whether they potentiate disease.

### Primary Sclerosing Cholangitis

Approximately 2% of patients with IBD has concomitant chronic inflammation of the bile ducts [73], called primary sclerosing cholangitis (PSC). PSC may also occur without signs of intestinal inflammation, but up to 80% of PSC patients was previously diagnosed with IBD (PSC-IBD). UC is the predominant disease phenotype, and it is assumed that all PSC patients will eventually be diagnosed with IBD [74]. PSC is diagnosed through imaging techniques such as endoscopic retrograde cholangiography (ERC). The hallmark characteristic of PSC is the formation of strictures around bile ducts, thereby causing cholestasis. One of the hypotheses for development of PSC is that lymphocytes show aberrant homing, i.e., exert immune-regulating functions in the liver rather can migrating towards the intestine [75].

In PSC, the flow of bile is reduced, which allows for microbial translocation into the bile ducts. This translocation was previously shown to associate with more rapid progression of disease [76]. Concerning fungal translocation, one study described that *Candida* species (*C. albicans, C. glabrata, C. tropicalis*) were found in bile of approximately 12% of PSC patients (*n* = 8, *N* = 67) [77]. Patients with biliary *Candida* spp. had elevated levels of the serum inflammation marker C-reactive protein (CRP) compared to *Candida*-negative PSC patients. In this cross-sectional study, three patients were treated with antifungal medications, which led to a mild reduction in liver inflammation [77]. A retrospective analysis of 150 PSC patients indicated that 20% has experienced either transient or persistent biliary candidiasis (i.e., a single *versus* consecutive positive cultures), which significantly reduced transplantation-free survival and an increased frequency of bile duct cancer [78]. One of the risk factors indicated for biliary candidiasis was the number of ERC examinations, hinting towards a possible contamination or aided translocation of microbes. As bile is notoriously difficult to collect without duodenal contamination, the findings on cultivable *Candida* spp. should be interpreted with caution.

Two recent studies have focused on describing the fecal mycobiota profiles of PSC patients instead. In the first study by the group of Sokol, fungal compositions of four patients groups (PSC only, PSC-IBD, IBD only, healthy volunteers; *N* = 112) were determined using ITS2-based metabarcoding [79]. *Candida* spp. was among the most abundant genera, but abundances hereof were equal between the four investigated groups. In disagreement with these results, an independent study (*N* = 109 individuals) showed significantly elevated abundance of *Candida* spp. in PSC patients compared with UC patients [80]. Since *Candida* spp. has immunogenic properties related to development of Th17 responses, the authors hypothesize that elevated abundance of *Candida* spp. in PSC may underlie previously observed aberrant immune response [81, 82].

### Irritable Bowel Syndrome

The functional bowel disorder irritable bowel syndrome (IBS) affects approximately 10% of the general population. The main symptom of IBS is abdominal pain related to (altered patterns of) defecation. The diagnosis is currently based on the Rome IV criteria, which state that (i) structural or biochemical alterations of the gastrointestinal tract should be excluded, and (ii) this should be combined with symptoms of IBS for a duration of at least six months [83]. Several factors contribute to the origin of IBS, including early-life stress, acute gastrointestinal infections, and psychological disease [84, 85]. The pathophysiological mechanism behind the characteristic abdominal pain is not fully understood yet, but the fungal gut composition likely contributes to the etiology of this disorder [86].

A large subset of patients experiences enhanced sensitivity to intestinal stimuli [87]. This so-called visceral hypersensitivity is, in part, driven by stress-related signaling, intestinal mast cell activation, and enhanced neuronal activation of intestinal nerves. In a rodent model of visceral hypersensitivity, the gut mycobiome was shown to associate with sensitivity status [17]. Here, the maternal separation model was used to study this enhanced sensitivity. In this model rat pups are predisposed by separating them from their mothers on postnatal days 2–14 for three hours each day. The rats are then conventionally raised until adult age, at which they are exposed to an acute stressor called water avoidance stress. The combination of early-life predisposition and adult stress evokes enhanced sensitivity towards colorectal distension. Control rats that did not undergo maternal separation (non-handled) do not show visceral hypersensitivity [88]. Using this model, Botschuijver et al. described a role for the gut mycobiome in visceral hypersensitivity, by showing that antifungal treatment in MS rats prevented and reduced visceral hypersensitivity [17]. The underlying pathophysiological mechanisms depended on Dectin-1/Syk signaling. In addition, the mycobiome composition of patients with IBS (*n* = 19 hypersensitive, *n* = 20 normosensitive) was described using ITS1-based metabarcoding. The composition hereof was found to be less diverse compared to that of healthy volunteers (*n* = 20) based on the Shannon diversity index. In all groups, either *S. cerevisiae* was the most abundant species, followed by *C. albicans*. The combined fraction of *S. cerevisiae* and *C. albicans* was more abundant in patients with IBS than healthy volunteers (HV), regardless of their sensitivity status.

The gut mycobiome of patients with IBS has been assessed by a few other research groups. Das et al. investigated fecal samples of 80 IBS patients and 64 HVs and indicate that, based on ITS1-based metabarcoding, the genus *Candida* is among the most prevalent genera [21]. While no differences in alpha diversity were observed between IBS and HV, the general compositions of these two groups were found to be different based on principle component analysis of Bray–Curtis distances. Moreover, several operational taxonomic units (OTUs) were differentially abundant between the two groups, of which two OTUs were significantly elevated in patients with IBS. One OTU related to *Candida* spp., and the other one to *C. albicans* [21]. The differential abundance of *C. albicans* was not observed in a study by Hong et al. where ITS2-based metabarcoding was used to describe the gut mycobiome of diarrhea-predominant IBS patients (*n* = 55) and HVs (*n* = 16). *Candida* spp. was the most abundant genus in the mycobiome profiles, and *C. albicans* abundance correlated significantly with self-reported abdominal bloating and the psychological comorbidity anxiety [19]. Plausible mechanisms for the observed associations were not provided, and it remains to be investigated whether altered fungal compositions or *Candida* spp. abundance contribute to (abdominal pain in) IBS.

Two studies have been performed up to date in which both the mycobiota composition as well as cultivable fungi were assessed. Sciavilla et al. observed a decreased number of OTUs in fecal samples of IBS patients (*n* = 20) compared with HVs (*n* = 18) and different mycobiota compositions based on Bray–Curtis dissimilarities [22]. While the metabarcoding-based abundance of *Candida* spp. in these fecal samples was not described, *C. albicans* (IBS and HV) was the most frequently observed fungal isolate in the culture-dependent approach followed by *C. parapsilosis* (IBS and HV) and *C. glabrata* (HV). The *C. albicans* isolates showed clonal expansion within individuals, but isolates originating from different patients were unrelated. Additionally, the *C. albicans* isolates were screened on various virulence-associated traits and growth rates, which resulted in enhanced virulent potential of IBS isolates based on hyphal formation and agar penetration rates [22]. The second study focused on fecal fungi in relation to visceral hypersensitivity. ITS1-based metabarcoding showed very few differences in composition or abundance of *C. albicans* between patients normo- and hypersensitive patients (*n* = 8 in each group). However, since previous research had indicated a fungal contribution to visceral hypersensitivity [17], it was hypothesized that functional capacities of the fungal community may contribute to altered colorectal sensitivity. The most frequently cultured species was again *C. albicans*, and isolates showed genetic variation within and between individuals [24]. Interestingly, isolates of hypersensitive IBS patients partially clustered together. Phenotypic characteristics including adherence to epithelial cells, enzyme release, and gene expression alterations during yeast-to-hyphae transition (*ECE1, ALS3*, and *SAP2*) were observed between the strains. No clear-cut association was observed between virulence-associated traits and visceral sensitivity status, in part due to small group sized in this study [24]. Further research should indicate whether the virulent capacity of *C. albicans* strains indeed contribute to altered sensitivity status and abdominal pain in IBS.

In conclusion, multiple reports indicate that *Candida* spp. and *C. albicans* are more abundantly present in fecal samples of patients with IBS. Besides, culture-dependent techniques have shown a wide phenotypic variation of *C. albicans* derived from IBS patients’ fecal material. Whether this is directly related to IBS-associated abdominal pain and/or visceral hypersensitivity remains to be determined in future studies.

## Challenges and Recommendations Regarding Studies of Intestinal *Candida* spp.

The knowledge on *Candida* spp. in the gastrointestinal tract is rapidly increasing but not yet fully understood. In this section, we will discuss several technical and biological challenges that limit the advancement of knowledge in this field.

### Technical Variation Among Sequencing- and Culture-Based Techniques

A major technical issue lies in describing the composition of the human gut mycobiota community, whether that is done by DNA- or culture-based approaches. Concerning DNA-based techniques, protocols for sequencing techniques often vary between labs, both on the aspect of wet lab and bioinformatics analyses, and the method of DNA isolation alone has great impact on the isolation performance and therefore the subsequent results [89]. Many commercial kits for determination of the bacterial gut profile are not aimed at proper retrieval of fungal DNA as these cells require a harsher extraction, leading to underestimations of fungal loads and possibly a bias towards fungal cells that are easier to lyse. Hereafter, the fungal composition is most commonly determined based on sequencing of the ITS regions. Up to this point, no long-read based approach commercial kits are available which limits identification of species. Thus, while both ITS1 and ITS2 metabarcoding are widely observed in literature, different profiles arise from sequencing of ITS1 vs. ITS2 regions. ITS1 shows more variation. Some of the technical challenges hide in the amplification processes preceding high-throughput sequencing. There are two main considerations that should be taken into account. Firstly, fungal DNA in fecal of mucosal samples is sparsely present. In order to determine the fungal compositions, multiple amplification steps may be necessary. Secondly, and related to this first observation, it should be considered that the length of the sequenced ITS region may vary, thereby always introducing an amplification bias. While this cannot be circumvented, these points should raise caution in interpretation of these results.

Another, but more costly, approach would be subjecting the obtained (DNA) samples to metagenomics sequencing. This shotgun sequencing approach is broadly used in research for the bacterial communities, but several bioinformatics tools are now arising to detect eukaryote- or fungal-specific sequences in these datasets such as HumanMycobiomeScan and EukDetect [90, 91]. The ability to extract fungal data from metagenomics sequencing was previously shown in the two CRC-related studies [71, 72], and this may thus open possibilities to reuse and further analyze fungal composition and functioning in existing datasets.

The determination of fungal communities is dependent on alignment of obtained data to references databases. Multiple references sources are available, but since not all fungi have been described or discovered yet, there are continuous gaps in these databases. Taxonomic information is missing for many of the obtained sequences, and if any classification is present, there often is no identification on the lower taxonomic levels. Thus in many studies, authors choose to report their findings on genus level rather than species level. In case of the genus *Candida* for example this is troublesome for further conclusions. As mentioned before, this genus has over 150 members all with greatly varying functional and pathogenic capabilities [8], and moreover, a number of members within this genus are less related to the other members [9]. We thus highly recommend to investigate mycobiota compositions below the genus level, especially for the genus *Candida.* Future expansions of genetic databases shall allow for more precise determination of fungal sequences and identities.

Similar to the lack of taxonomic classification or identification, differences between fungal strains within individuals cannot be detected using ITS metabarcoding nor by Sanger sequencing. This sub-species variability may be important in disease as previously described for IBD and IBS [22, 24, 28]. More specialized techniques such as Amplified Fragment Length Polymorphism fingerprinting (AFLP), Random Amplified Polymorphic DNA (RAPD), microsatellite typing or whole genome sequencing (WGS) are needed to properly describe these variations. In order to do so, the fungal cells must first be obtained from the sample of interest. Culture-based techniques provide a decent starting point to investigate which fungi could survive the harsh environment of the human intestine. However, the observed diversity in such experiments if often very low as culture-based techniques obscure fungi that are hard to culture (e.g., nutrients, oxygen levels, temperature variations, dependencies on metabolites of other microbes). For example, sequencing-based techniques indicate that the genus *Candida* is among the most prevalent genera in the human intestine, and this is also seen in culture-based studies. However, *Candida* spp. is fairly easy to culture as opposed to e.g., *Malassezia* spp., which is often also observed among the more abundant species. One way to overcome these issues is by culturing the sample of interest on multiple culture media and under different conditions, although this is no guarantee to successful culture of living gut fungi.

### Biological Challenges: Strain Variability, Functionality and Causality

The gut mycobiome is subject to many variations and the composition can heavily be influenced by external factors. Of several fungi, it is remains elusive why they are found in intestine-derived samples. As they could well be passers-by in the intestine (such as the airborne *Cladosporium* spp.) or food-derived (e.g., *S. cerevisiae*, *Penicillium roquefortii, Agaricus bisporus*). And even if these cases are excluded, the question remains whether the fungal species is able to survive within the intestinal environment and contribute to this ecosystem. Thus, diet, consumption of carbohydrates, and geographical locations have been described as being able to alter the gut mycobiome composition. Aside from these external factors, the gut mycobiome appears to be less stable than the bacterial counterpart. In a study of 24 longitudinal samples, less than 20% of fungi were detected at both time-points [92]. This observation was confirmed in the large study of the gut mycobiome for the HMP project. Here, the within-individual variation of the gut mycobiome was similar to that observed between the included individuals [26]. This intrinsic variation of the fungal composition thus further complicates research regarding the role of fungi in health and disease.Another key limitation of metabarcoding studies is the lack of information on the functional capacities of the gut mycobiome as determining the pool of ITS sequencing in a complex sample rather provides information about the composition. Functional capacities, such as invasiveness of fungi *versus* commensalism, and release of enzymes or metabolites cannot be assessed through metabarcoding. In order to study the functional potential, either analytical chemical techniques are necessary (e.g. mass spectrometry-, chromatography-based) or alternatively, fungi could be cultured and next studied in appropriate in vitro or in vivo settings. Studies using this methodology have shown substantial inter-individual variation of *C. albicans* enzyme release, virulence-related traits, and associations with intestinal inflammation [22, 24, 28]. With current technologies, culture of fecal *Candida* spp. remains an essential step in determination of the pathogenic potential of single strains.

Unfortunately, the culture-based approaches reduce the dimensionality of the data and could introduce bias in follow-up investigations. In this light, it is worth mentioning that the studies regarding the gut mycobiome are rarely describing causality, but merely associative observations in relation to health or disease. Describing causal relations in the human context remains challenging as mechanistic studies are not possible from an ethical standpoint. While in vivo disease models allow for disease-related studies to some extent, it should be noted that murine models are never perfectly resembling the human (diversity of) pathophysiological processes, and that the intestine of a laboratory mouse usually barely contains any fungi. Relating back to the importance of immune system development under influence of the intestinal mycobiome, all experimental mice should ideally be inoculated with a (standardized) consortium of fungi, e.g., as done by van Tilburg-Bernardes et al. [3], especially since mono-colonization using a single strain of *C. albicans* is often not successful [93]. Thus, in vivo studies regarding the gut mycobiome in general and strain diversity specifically should be subjected to proper optimization.

In the field of gastroenterology, the existence of sub-species variability in yeast has recently started gaining attention. It has now been shown that strain variability occurs within the intestine of all individuals, irrespective of their health status (i.e., healthy, IBD, IBS) [22, 24, 28, 94]. This variation typically remains masked using common metabarcoding approaches, thereby explicitly requiring the additional step of culture-based techniques. Several articles describe that the altered genotypes occur both within and between individuals. Direct consequences for disease status were not yet clearly described, although Li et al. describe that more virulent strains associate with worsened intestinal inflammation [28]. While this is now relatively well described for *C. albicans*, such variation has to our knowledge not yet been described for non-albicans *Candida* species derived from intestinal or fecal samples. For *C. parapsilosis*, genetic variation of clinical isolates was previously described between different human locations (e.g., feces, blood, urine, and liquor), but was reported to be extremely low especially compared to *C. albicans* [95, 96]. Whether inter- and intra-individual genetic or phenotypic variation of *C. parapsilosis* contribute to intestinal diseases remains to be established. Future research should indicate whether similar patterns can be observed for other fungal species, and if or how this variation contributes to (intestinal) disease.

Taken together, the biological variation among intestinal *Candida* species and likely other yeast and fungi further complicates research on the role of the gut mycobiome. While metabarcoding now relatively easily profiles a complete view of the gut fungal community, the functional aspects cannot be deducted from such analyses. Culture-based techniques may thus, performed next to metabarcoding studies, provide additional information on the contribution of fungi in pathophysiological processes underlying intestinal disease.

#### Trans-Kingdom Interactions in Health and Disease

Although multiple associations have been shown between disease and the intestinal fungal community, it is unlikely that the fungal component solely contributes to pathophysiologic processes. The intestinal microbial community consist for the majority of bacteria, and interactions between the different kingdoms through metabolic or physical interactions are also occurring in health and disease. These interactions have been described before in light of multiple disease states as also reviewed by Zhang et al. [7]. In relation to IBD, Leonardi et al. observed significant correlations between abundance of *Candida* spp. and bacterial genera such as *Ruminococcus* and *Sutterella,* and the authors therefor hypothesize that *Candida* spp. may have an influence on shaping of the bacterial composition [97]. Moreover, Sokol et al. previously investigated associations between fungal and bacterial abundances. Multiple of these interactions were observed, but most notably, a larger number of correlations was described for UC patients than for CD or healthy volunteers [18]. Hong et al. performed similar analyses for IBS, but observed that the healthy gut microbes showed more and stronger correlations than patients with IBS [19]. Such interactions between fungi and bacteria can also be modelled in silico, as was performed in an investigation of *C. albicans* metabolism and interactions with other organisms. Over 900 bacterial-fungal interactions were assessed in so-called metabolic models. Several bacteria were associated with alteration of *C. albicans* levels in feces. For example, the authors confirmed these suggested findings in human fecal samples, and conclude that *Alistipes putredinis* may prevent high levels of *C. albicans* in feces [98]. As the number of interactions between bacteria and fungi increases, we would suggest to assess at least both the bacterial and fungal component in intestine-derived tissues, assess which relations between kingdoms occur within the framework of a certain disease, and how these may contribute to the intestinal disease.

## Conclusion

In the human gut fungal community, *Candida* spp. is the most frequently observed fungal genus by culture-dependent and –independent techniques, with as leading species *C. albicans, C. parapsilosis,* and *C. glabrata.* Given the opportunistic nature of *Candida* spp., this genus has been associated with prematurity-related intestinal diseases such as NEC, and adult diseases including IBD and IBS. In addition, intestinal *Candida* spp. can be linked to CRC and PSC as well, and it is likely that this genus may be involved in multiple other diseases beyond the GI tract. At present, several challenges exist regarding the study of Candida spp. in the human intestine, with as foremost difficulty being technical limitations of metabarcoding, as well as the physiological biological variation among strains of *C. albicans*. Moreover, study models of intestinal disease never fully capture the vast heterogeneity of pathological mechanisms in disease, and the performed work therefore often remains of a descriptive nature. While associations between the gut mycobiome and intestinal disease are more often confirmed, future research should point out whether fungi have a functional role in development or worsening of intestinal disease, and whether intestinal *Candida* spp. or fungi in general could be a target for alleviation of disease.
